# Global distribution and temporal analysis of R70Q/H oncogenic mutations in hepatitis C virus subtype 1b core protein

**DOI:** 10.3389/fcimb.2025.1619865

**Published:** 2025-09-02

**Authors:** Gabriela T. M. Nunes, Natalia M. Araujo

**Affiliations:** Laboratory of Molecular Virology and Parasitology, Oswaldo Cruz Institute, FIOCRUZ, Rio de Janeiro, Brazil

**Keywords:** HCV, hepatocellular carcinoma, core protein mutations, geographic distribution, antiviral therapy, viral evolution

## Abstract

The hepatitis C virus (HCV) core protein is crucial in viral pathogenesis and hepatocarcinogenesis. Amino acid substitutions at position 70, particularly R70Q and R70H, are associated with an increased risk of hepatocellular carcinoma (HCC) and partial resistance to interferon-based therapy in genotype 1b infections. However, the global and temporal dynamics of these oncogenic mutations remain poorly understood. In this study, we analyzed 3,218 publicly available HCV subtype 1b core sequences to investigate the global distribution of R70Q/H mutations and their evolution across therapeutic eras. Our findings reveal notable regional disparities, with R70Q prevalence highest in Western Europe (77.4%) and Northern America (70.4%), while R70H was most frequent in Central America (45%). Temporal analysis of 1,351 dated sequences showed a significant decline in R70Q/H frequency during the pegylated interferon plus ribavirin era (2001–2010: 24%) compared to the conventional interferon period (1989–2000: 39%; *p* = 0.0081), followed by a resurgence in the direct-acting antivirals (DAAs) era (2014–present: 43%; *p* = 0.0183). These temporal shifts, including both the decline and resurgence, suggest a complex interplay between treatment-related selective forces, viral diversity, host factors, and possibly sampling bias. Our results underscore the need for regional molecular surveillance to guide HCC monitoring in HCV subtype 1b patients with R70Q/H mutations, even after viral clearance, and to inform targeted prevention strategies in high-prevalence areas.

## 1 Introduction

Hepatitis C virus (HCV), a member of the *Flaviviridae* family, is an enveloped virus with a single-stranded, positive-sense RNA genome of approximately 9.6 kilobases in length (Choo et al., 1989; Moradpour et al., 2007). HCV replication is remarkably efficient, with estimates suggesting the production of up to 10¹² virions per day (Neumann et al., 1998). Combined with the absence of proofreading by the viral RNA-dependent RNA polymerase, this high replication rate contributes to extensive genetic variability. As of 2022, HCV is classified into eight genotypes and over 90 subtypes, with up to 30% nucleotide divergence between genotypes and about 15% between subtypes (ICTV, 2024). The World Health Organization (WHO) estimates that around 50 million individuals are chronically infected with HCV, with nearly one million new cases annually, leading to approximately 242,000 deaths in 2022, mainly due to liver cirrhosis and hepatocellular carcinoma (HCC) (WHO, 2024).

HCC is the most common type of primary liver cancer, accounting for 75–85% of all cases. Globally, liver cancer is the sixth most frequently diagnosed cancer and the third leading cause of cancer-related deaths, with an estimated 866,000 new cases and 758,000 deaths each year (Sung et al., 2021; Ferlay et al., 2024). Chronic HCV infection is a leading risk factor for HCC, especially in cirrhotic patients. Even after successful viral eradication with direct-acting antivirals (DAAs), which achieve cure rates exceeding 95%, a residual HCC risk persists, particularly in those with advanced liver disease or harboring viral variants with certain HCV core mutations (Akuta et al., 2011, 2015; Ogata et al., 2018; Luan et al., 2023). In fact, among cirrhotic patients, those with HCV face the highest likelihood of developing HCC, underscoring the need for long-term monitoring even post-treatment (Fattovich et al., 2004). HCV-associated oncogenesis involves not only chronic inflammation and increased hepatocyte turnover but also direct interactions between viral proteins and host cellular pathways. The HCV core protein plays a key role in this process, modulating cell proliferation, apoptosis, transcription, and immune responses (Yao et al., 2001; Zhu et al., 2001; Sato et al., 2006; Khaliq et al., 2011). Notably, among the mutations in the core region, substitutions at position 70, particularly arginine to glutamine (R70Q) and arginine to histidine (R70H), have been widely associated with increased HCC risk in genotype 1b infections (El-Shamy et al., 2013; Araujo et al., 2014; Akuta et al., 2015; Ogata et al., 2018), and interferon therapy resistance (Kurbanov et al., 2010; Funaoka et al., 2011; Kichatova et al., 2018), underscoring the clinical importance of identifying and monitoring these variants.

Given the geographic variability in HCV genotypes, host genetic backgrounds, and disparities in healthcare access, differences in mutation prevalence and oncogenic potential across regions are to be expected. This study aims to investigate a large dataset of publicly available HCV genotype 1b sequences from multiple regions worldwide, with a particular focus on mutations at position 70 of the core protein. By analyzing their global distribution and temporal trends across therapeutic eras, we aim to identify regional prevalence patterns that may provide important insights into HCC risk, thereby guiding public health strategies tailored to local epidemiological contexts.

## 2 Materials and methods

### 2.1 HCV genomic sequences

HCV genotype 1 core region sequences (n = 6,075) available in the Los Alamos HCV Database (https://hcv.lanl.gov/content/sequence/HCV/ToolsOutline.html) were retrieved from a precomputed dataset containing aligned sequences in FASTA format, downloaded on 9 October 2024. Inclusion required coverage of the complete core coding region. This criterion improves the reliability of reported subtype annotations and enables unambiguous residue calling and consistent alignment at position 70. Metadata on sequence origin and deposition date were extracted from existing annotations in the Los Alamos database. After filtering for sequences with available information on subtype 1b and country of origin, 3,218 sequences were selected for the study. Sequences were further grouped into 21 geographic subregions according to the United Nations Statistical Division (UNSD) M49 classification (https://unstats.un.org/unsd/methodology/m49/#geo-regions). For the temporal analysis, a total of 1,351 subtype 1b sequences were filtered from the dataset based on the availability of collection date information.

### 2.2 Mutational analysis

Sequence alignments were analyzed using MEGA11: Molecular Evolutionary Genetics Analysis version 11 (Tamura et al., 2021). An Excel dataset was compiled containing HCV subtype 1b sequences, the amino acid at core position 70, the corresponding country of origin, and the collection date information for each sequence (**Supplementary Table S1**).

### 2.3 Statistical analysis

Statistical analysis was performed using SPSS statistical software package version 23.0 (IBM SPSS Inc., Chicago, IL, United States). Frequencies were compared using the chi-squared test or Fisher’s exact test. A *p-*value < 0.05 was considered statistically significant.

## 3 Results

**Figure 1:**
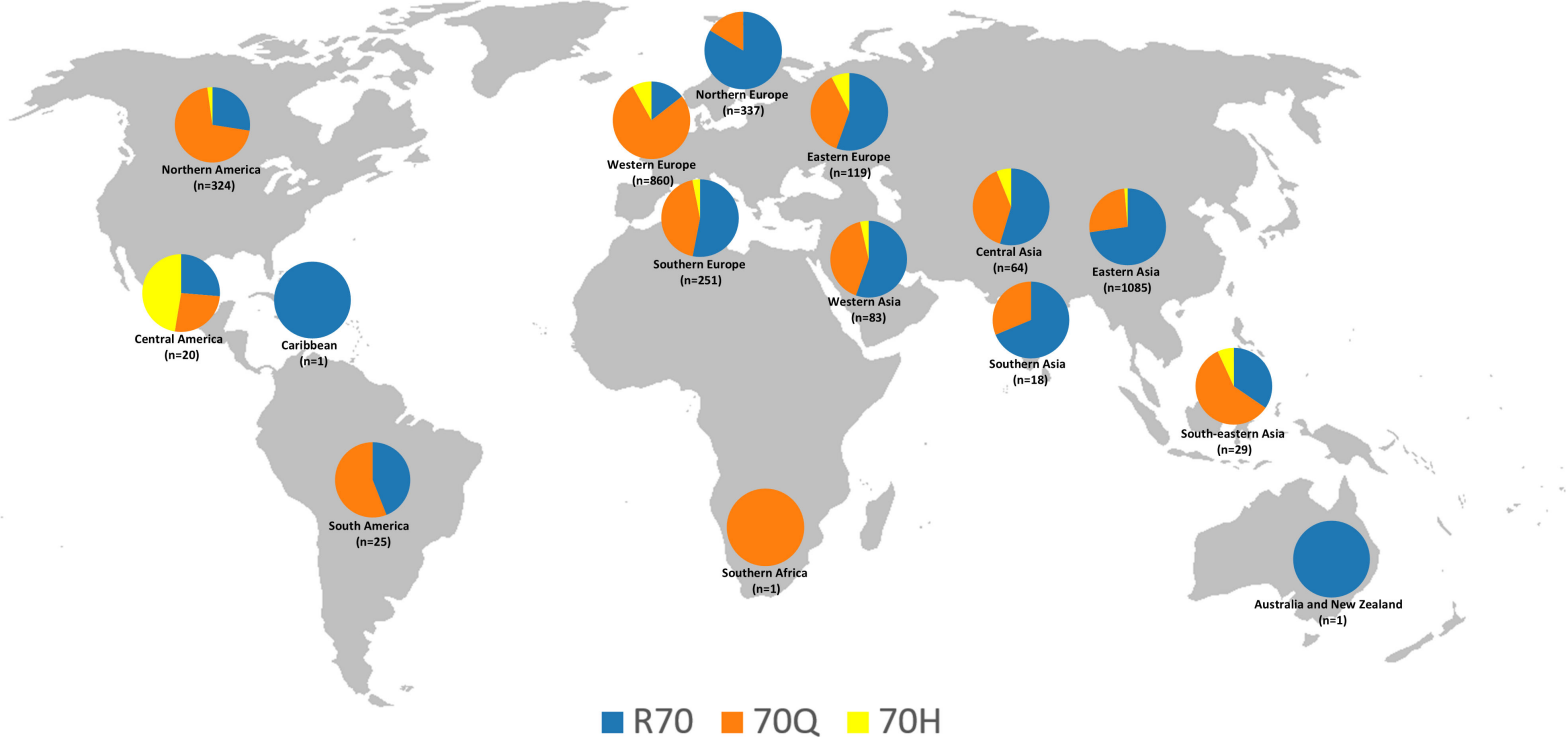
Geographic distribution of the frequencies of amino acids R, Q, and H at position 70 of the HCV subtype 1b core protein, based on 3,218 sequences from the Los Alamos database. The map was reconstructed using a base image from Wikimedia Commons (https://commons.wikimedia.org/wiki/File:BlankMap-World-noborders.png); this figure is similar but not identical to the original and is intended for illustrative purposes only.

Based on the data presented in **Table 1**, a detailed analysis of the amino acid substitutions at position 70 of the HCV core protein (subtype 1b) reveals pronounced regional disparities in the prevalence of R70Q and R70H mutations. These findings suggest the existence of distinct epidemiological landscapes and potential geographic hotspots for oncogenic viral variants. Among the 3,218 sequences analyzed globally, the R70Q mutation was observed in 46.1% of sequences, underscoring its widespread distribution, whereas R70H was identified in only 3.9%, indicating a more restricted and regionally concentrated pattern (**Table 1**). A map displaying the distribution of amino acid frequencies across different geographical subregions is shown in **Figure 1**.

**Table 1:** Frequency of amino acids R, Q, and H at position 70 of HCV subtype 1b core protein across geographic regions.

	Residue^a^			
Geographic region	R (%)	Q (%)	H (%)	Others^b^ (%)
Africa (n=1)	0 (0)	1 (100)	0 (0)	0 (0)
Southern Africa (n=1)	0 (0)	1 (100)	0 (0)	0 (0)
Americas (n=370)	106 (28.6)	247 (66.8)	16 (4.3)	1 (0.3)
Central America (n=20)	5 (25)	5 (25)	9 (45)	1 (5)
Northern America (n=324)	89 (27.5)	228 (70.4)	7 (2.2)	0 (0)
South America (n=25)	11 (44)	14 (56)	0 (0)	0 (0)
Caribbean (n=1)	1 (100)	0 (0)	0 (0)	0 (0)
Asia (n=1279)	886 (69.3)	360 (28.1)	24 (1.9)	9 (0.7)
Central Asia (n=64)	35 (54.7)	25 (39.1)	4 (6.3)	0 (0)
Eastern Asia (n=1085)	784 (72.3)	279 (25.7)	15 (1.4)	7 (0.6)
South-eastern Asia (n=29)	10 (34.5)	17 (58.6)	2 (6.9)	0 (0)
Southern Asia (n=18)	11 (61.1)	5 (27.8)	0 (0)	2 (11.1)
Western Asia (n=83)	46 (55.4)	34 (41)	3 (3.6)	0 (0)
Europe (n=1567)	603 (38.5)	876 (55.9)	87 (5.6)	1 (0.1)
Eastern Europe (n=119)	66 (55.5)	44 (37)	9 (7.6)	0 (0)
Northern Europe (n=337)	281 (83.4)	55 (16.3)	0 (0)	1 (0.3)
Southern Europe (n=251)	132 (52.6)	111 (44.2)	8 (3.2)	0 (0)
Western Europe (n=860)	124 (14.4)	666 (77.4)	70 (8.1)	0 (0)
Oceania (n=1)	1 (100)	0 (0)	0 (0)	0 (0)
Australia and New Zealand (n=1)	1 (100)	0 (0)	0 (0)	0 (0)
Total (n=3218)	1596 (49.6)	1484 (46.1)	127 (3.9)	11 (0.3)
*p*-value	0.0001	0.0001	0.0001	0.0103

a R (arginine) is wild-type; Q (glutamine) and H (histidine) are HCC-associated variants.

b Other amino acid variants not associated with HCC.

In the Americas, the overall prevalence of R70Q was notably high (66.8%), driven primarily by Northern America, where the mutation was present in 70.4% of sequences. This suggests a substantial circulation of viral strains associated with increased oncogenicity. Conversely, Central America exhibited a markedly lower R70Q prevalence (25%) but had the highest global frequency of R70H (45%; exclusively represented by sequences from Mexico, as classified in this region by the UN geoscheme), possibly reflecting the emergence or persistence of unique viral lineages. South America presented intermediate R70Q levels (56%) and no detection of R70H, while the Caribbean, represented by a single sequence, retained the wild-type R at position 70.

Asia, the continent contributing the largest dataset (n=1,279), demonstrated substantial subregional heterogeneity. South-eastern Asia showed a high frequency of R70Q (58.6%) and a moderate presence of R70H (6.9%), indicating a potential hotspot for both mutations. Central Asia (R70Q: 39.1%; R70H: 6.3%) and Western Asia (R70Q: 41%; R70H: 3.6%) also exhibited noteworthy mutation frequencies. In contrast, Eastern Asia, the most extensively sampled subregion (n = 1,085), displayed markedly lower rates of both R70Q (25.7%) and R70H (1.4%), suggesting the influence of distinct host-virus interactions, treatment histories, or evolutionary constraints. Southern Asia exhibited moderate R70Q prevalence (27.8%) and no evidence of R70H.

Europe revealed some of the most divergent intra-continental patterns. Western Europe emerged as a prominent hotspot, with the highest global frequencies of both R70Q (77.4%) and R70H (8.1%), reflecting a significant burden of oncogenic variants. In contrast, Northern Europe exhibited a strong predominance of the wild-type residue (R: 83.4%), indicating limited circulation of mutated strains. Eastern and Southern Europe presented intermediate R70Q frequencies (37% and 44.2%, respectively), accompanied by moderate levels of R70H (7.6% and 3.2%).

The representation of Africa and Oceania in the dataset was extremely limited, preventing the formulation of robust conclusions regarding the distribution of mutations in these regions. Only one sequence was available from Africa, sourced from Southern Africa, which harbored the R70Q mutation. Oceania was represented by a single sequence from Australia, which retained the wild-type R residue.

**Figure 2:**
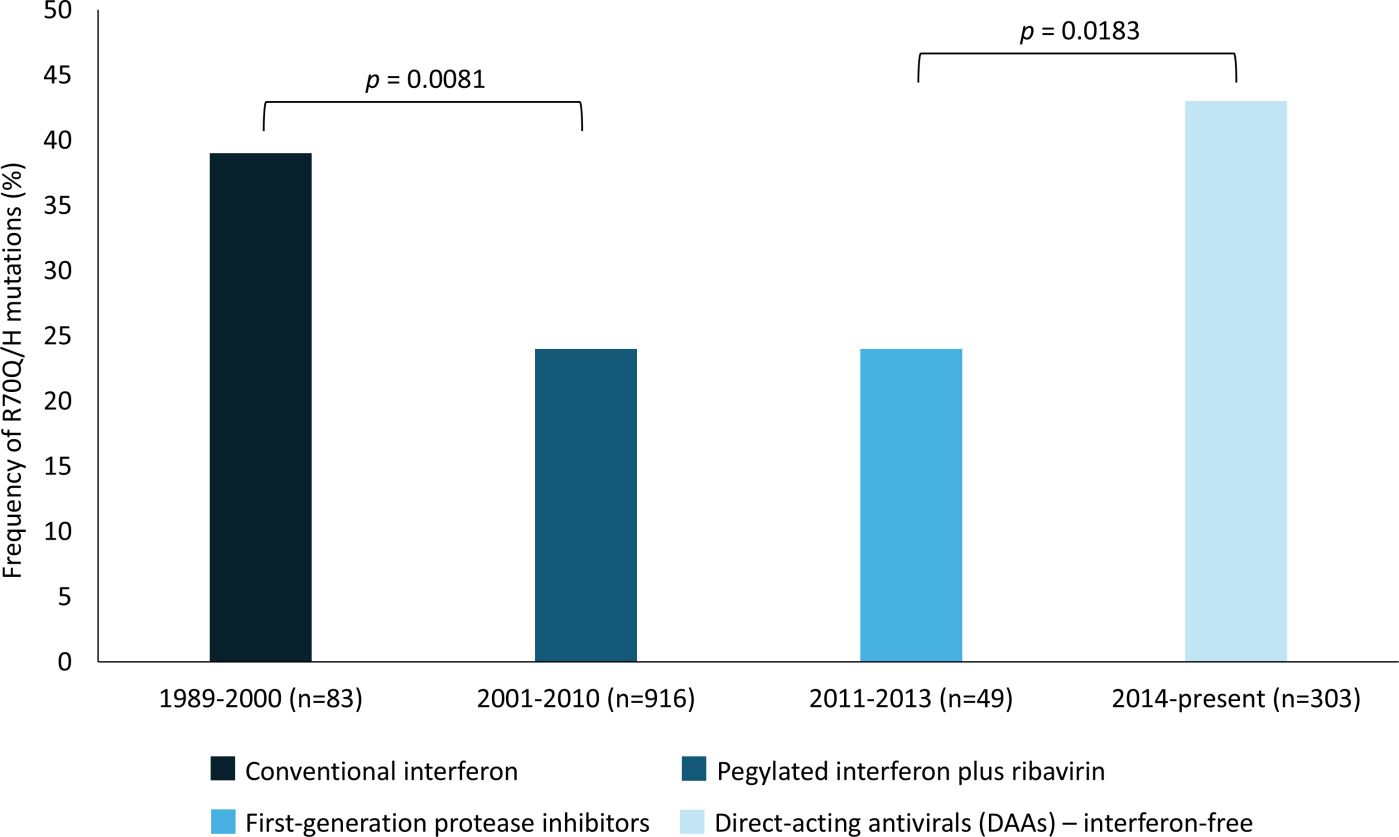
Prevalence of R70Q/H mutations in HCV-1b core sequences (n=1,351) over time, stratified by treatment era. Significant differences between eras are indicated (*p*-values). Treatment regimens are represented by color-coded bars: conventional interferon (1989-2000), pegylated interferon plus ribavirin (2001-2010), first-generation protease inhibitors (2011-2013), and DAAs interferon-free regimens (2014-present).

To evaluate the temporal distribution of the R70Q/H mutations in the core region of HCV subtype 1b, we analyzed a total of 1,351 sequences retrieved from the Los Alamos database that contained information on the year of sample collection. The sequences were grouped into four time periods corresponding to distinct therapeutic eras: 1989–2000 (conventional interferon therapy), 2001–2010 (pegylated interferon plus ribavirin), 2011–2013 (first-generation protease inhibitors), and 2014–present (DAAs, interferon-free regimens) (**Figure 2**). Our results revealed notable fluctuations in the prevalence of R70Q/H mutations across these periods. The proportion of sequences carrying the mutations was 39% in the 1989–2000 interval, followed by a significant decrease to 24% during 2001–2010 (*p* = 0.0081). The frequency remained stable at 24% during 2011–2013, then significantly increased to 43% from 2014 onwards (*p* = 0.0183).

## 4 Discussion

Multiple cohort studies and multivariate analyses have identified core position-70 variants as an independent predictor of increased HCC risk in patients with chronic HCV genotype 1b infection, both in those with active viremia and after viral eradication (Hu et al., 2009; El-Shamy et al., 2013; Akuta et al., 2015; Ogata et al., 2018). Although core position-91 substitutions have also been linked to HCC in some reports, the evidence is less consistent and often appears in combination with R70Q/H, suggesting a possible synergistic rather than independent effect (Sedeno-Monge et al., 2017). Given these data, position 70 was selected as the primary target for our study because it represents a well-established, independent viral marker of hepatocarcinogenesis risk, making it more suitable for focused clinical and epidemiological evaluation. Although R70Q/H has been detected in other HCV genotypes/subtypes such as 5a (Bhebhe et al., 2019) and 1a and 3a (Araujo et al., 2014), the consistent association between these variants and an increased risk of HCC has been demonstrated only for subtype 1b. Therefore, our study focused exclusively on subtype 1b to investigate geographic and temporal patterns of R70Q/H with proven clinical relevance for HCC risk stratification. Future studies including genotypes beyond 1b may help to determine whether similar epidemiological and clinical implications exist in other viral backgrounds.

The analysis of HCV subtype 1b core mutations reveals complex geographic and temporal patterns in the prevalence of R70Q and R70H. While these mutations are globally distributed, their frequencies vary markedly across regions and therapeutic eras, reflecting a dynamic interplay between antiviral selective pressures, viral evolution, and regional epidemiological contexts.

Geographically, the high prevalence of R70Q in Western Europe (77.4%) and Northern America (70.4%) aligns with the early and widespread adoption of interferon-based therapies in these regions. *In vitro* and clinical studies suggest that R70Q may confer partial resistance to interferon antiviral effects (Kurbanov et al., 2010; Funaoka et al., 2011; Kichatova et al., 2018), and its accumulation likely reflects prolonged selection pressure from suboptimal interferon monotherapy regimens. Conversely, East Asia showed the lowest frequencies of both R70Q (25.7%) and R70H (1.4%), despite contributing the largest number of sequences. At first glance, this may suggest a relatively conserved viral population or differing evolutionary pressures. However, a closer country-level analysis revealed substantial heterogeneity within the region. Japan, which accounted for the majority of sequences (n=539), exhibited a markedly higher frequency of mutant variants (51%). This finding is consistent with the country’s long-standing and widespread use of interferon-based therapies since the early 1990s (Akuta et al., 2011, 2015), which may have contributed to the selection of less responsive variants such as R70Q. Conversely, in China, a country with a comparable number of sequences (n=532), 97% were wild-type strains. This disparity may be related to historical differences in treatment accessibility and timing of therapy rollout, with China experiencing more limited and gradual access to interferon-based therapies compared to Japan. As a result, the selective pressure for the emergence of R70Q/H variants may have been lower. Additionally, the predominance of distinct HCV subtype 1b lineages with different fitness characteristics could also have contributed to the maintenance of wild-type strains in the Chinese population. Interestingly, Central America (here including all sequences from Mexico, according to the UNSD M49 classification) displayed a unique mutational profile, with the highest global frequency of R70H (45%) and a relatively low prevalence of R70Q (25%). This distinctive pattern may indicate the circulation of regionally confined viral lineages and warrants further investigation. Overall, these geographic differences likely reflect distinct viral evolutionary trajectories shaped by a complex interplay of factors, including host genetic backgrounds, HCV transmission dynamics, and the timing and intensity of antiviral treatment programs.

The temporal analysis of R70Q/H mutations in HCV subtype 1b sequences revealed dynamic shifts in their prevalence across distinct therapeutic eras. The significant decrease in frequency observed during the pegylated interferon plus ribavirin era (2001–2010), compared with the conventional interferon period (1989–2000), may reflect the higher antiviral potency of the optimized interferon formulation combined with the additive effect of ribavirin. Together, these agents could have exerted stronger selective pressure against viral variants harboring R70Q/H. The frequency of these mutations then remained stable during the first-generation protease inhibitor era (2011–2013), likely because these drugs, although representing a therapeutic advance, do not target the HCV core protein and continued to be administered alongside peg-interferon and ribavirin, thereby maintaining a similar selective environment. Unexpectedly, a significant resurgence of R70Q/H mutations was observed in the interferon-free era (2014–present), reaching the highest frequency among all periods analyzed. Since DAAs do not target the HCV core protein and these mutations do not confer resistance to them, this increase suggests that other evolutionary or clinical mechanisms may be at play. One possibility is that the removal of peginterferon/ribavirin therapy lifted prior selective constraints, allowing previously suppressed variants to re-emerge. Alternatively, it is also important to consider the potential influence of sampling bias. In the DAA era, sequencing efforts may have disproportionately focused on patients with advanced liver disease or HCC, clinical contexts where core mutations like R70Q/H are more commonly investigated. This overrepresentation could artificially inflate the observed prevalence of these variants in public databases. This mechanistic explanation, however, remains hypothesis-based. Given the significant regional differences in mutation prevalence, uneven sample sizes across time periods, and the likelihood of overrepresentation of certain clinical contexts in public databases, alternative or additional factors cannot be ruled out.

The seemingly contradictory patterns observed in the epidemiology of HCV R70Q/H mutations, including geographic accumulation in regions with prolonged interferon use versus temporal decline during optimized therapy, demonstrate that their distribution cannot be explained by any single factor. Rather, geographic and temporal patterns are shaped by overlapping yet distinct forces. From a geographic perspective, regions with extensive histories of interferon monotherapy, such as Japan, Northern America and Western Europe, show significant mutation accumulation, while areas that rapidly adopted DAAs or had limited historical access to interferon-based therapy maintain lower frequencies. This highlights the critical role of therapeutic history in shaping viral evolution. Beyond treatment effects, viral lineage diversity contributes substantially, with founder effects and localized strain circulation creating distinct regional hotspots, exemplified by the unusually high prevalence of R70H in Central America. Furthermore, host-pathogen interactions play a key role, as mutations like R70Q may confer additional fitness advantages beyond interferon resistance, including enhanced immune evasion capabilities and activation of oncogenic pathways (Khaliq et al., 2011). These multifaceted benefits could explain why such mutations persist in viral populations even in the DAAs era, despite the absence of direct selective pressure from these newer antiviral regimens. Together, these factors (therapeutic history, viral genetic diversity, and host-pathogen dynamics) form an integrated framework that explains both the geographic variability and temporal fluctuations observed in R70Q/H mutation patterns worldwide.

Several limitations of this study should be acknowledged. First, our analysis relied on publicly available sequences, which may not fully capture the global diversity of circulating HCV strains, especially in underrepresented regions such as Africa and Oceania. Second, the lack of detailed clinical metadata (e.g., fibrosis stage, prior treatment history, or HCC status) limits our ability to directly correlate mutational profiles with patient outcomes. Third, the dated sequences used for temporal analysis are unevenly distributed across geographic regions and therapeutic eras, and significant regional differences in mutation prevalence, together with variable sample sizes, may influence the observed temporal patterns. Moreover, public databases in the DAA era may overrepresent patients with advanced liver disease or HCC, potentially biasing prevalence estimates for R70Q/H in recent years. Finally, treating each sequence as an independent isolate may overlook the possibility of sampling from clonal populations, potentially introducing redundancy. However, the large dataset analyzed here likely mitigates these limitations to some extent.

Taken together, the observed regional and temporal patterns in the distribution of R70Q and R70H mutations in the HCV subtype 1b core protein provide valuable insights into the evolutionary dynamics of oncogenic variants. The differential distribution of these mutations suggests that public health strategies, including post-sustained virological response surveillance and HCC screening, should consider the local mutational landscape when tailoring monitoring efforts.

## Data Availability

The original contributions presented in the study are included in the article/**Supplementary Material**. Further inquiries can be directed to the corresponding author.

## Supplementary material

Supplementary Table 1 HCV subtype 1b core sequences dataset.
